# 3D Object Reconstruction from Imperfect Depth Data Using Extended YOLOv3 Network

**DOI:** 10.3390/s20072025

**Published:** 2020-04-03

**Authors:** Audrius Kulikajevas, Rytis Maskeliūnas, Robertas Damaševičius, Edmond S. L. Ho

**Affiliations:** 1 Department of Multimedia Engineering, Kaunas University of Technology, 51423 Kaunas, Lithuania; audrius.kulikajevas@ktu.edu (A.K.); rytis.maskeliunas@ktu.lt (R.M.); 2 Department of Applied Informatics, Vytautas Magnus University, 44404 Kaunas, Lithuania; 3 Faculty of Applied Mathematics, Silesian University of Technology, 44-100 Gliwice, Poland; 4 Department of Computer and Information Sciences, Northumbria University, Newcastle upon Tyne NE1 8ST, UK; e.ho@northumbria.ac.uk

**Keywords:** 3D scanning, 3D shape reconstruction, RGB-D sensors, imperfect data, hybrid neural networks

## Abstract

State-of-the-art intelligent versatile applications provoke the usage of full 3D, depth-based streams, especially in the scenarios of intelligent remote control and communications, where virtual and augmented reality will soon become outdated and are forecasted to be replaced by point cloud streams providing explorable 3D environments of communication and industrial data. One of the most novel approaches employed in modern object reconstruction methods is to use a priori knowledge of the objects that are being reconstructed. Our approach is different as we strive to reconstruct a 3D object within much more difficult scenarios of limited data availability. Data stream is often limited by insufficient depth camera coverage and, as a result, the objects are occluded and data is lost. Our proposed hybrid artificial neural network modifications have improved the reconstruction results by 8.53% which allows us for much more precise filling of occluded object sides and reduction of noise during the process. Furthermore, the addition of object segmentation masks and the individual object instance classification is a leap forward towards a general-purpose scene reconstruction as opposed to a single object reconstruction task due to the ability to mask out overlapping object instances and using only masked object area in the reconstruction process.

## 1. Introduction

One of the pressing issues in computer vision is three-dimensional (3D) object reconstruction, due to it becoming a core technology in numerous high-end industrial applications such as smart manufacturing, industrial automation and Industry 4.0 [1]. Moreover, there exists a wide variety of applications that would benefit from real time computer vision systems that are capable of fully reconstructing scenes, with most notable examples being an interactive medium such as virtual reality (VR) games and simulations [2], augmented reality (AR) applications or even in newest booming technologies such as extended reality (XR) [3]. Further examples for applications of such systems could include gesture [4,5] and posture [6] applications, indoor mapping [7], obstacle detection [8] recreating environments in movies or even digital forensics [9] to allow for crime scene recreation, robotics [10], teleconferencing [11] with the use of holograms and more. Therefore, we can safely assert that there is definitely a need for affordable, commercially viable solutions capable of providing real-time reconstruction capabilities available to the average user with as little complexity and barrier of entry, in terms of both financial investments and knowledge about the field, as possible.

As we cannot expect an average user to have the access to professional filming sets, mounting arrays of laser scanners capable of scanning the entirety of the room, in addition to the computing resources that would be required to stitch the data retrieved from multiple high-fidelity depth sensors, we need a solution that would meet or exceed the previous caveats. Therefore, we need a solution capable of working in real-time on a regular non-enthusiast grade workstation or even on a laptop. Furthermore, while we cannot expect the user to have a modern sensor array setup we can try to minimize the initial setup cost to a single depth sensor available in electronics stores or even in quite a few modern mid-tier and flagship phones. While solutions for scene reconstruction from a single depth sensor already exist, these solutions require incremental building per each frame [12,13]. This is done based on camera localization information and delta frames and in the scene reconstruction algorithms that make use of simultaneous localization and mapping (SLAM) [14]. To reliably fill all the holes in the areas that are occluded by other objects and even because of self-occlusion, we would have to scan the entirety of the object from all sides to have its full profile. Furthermore, incremental methods tend to underperform because of one principal flaw: changes in the scene can disrupt the mesh [15]. Making the applications in non-static real world scenes limited, where instead of the entirety of the view moving some objects can change their localization, or even suddenly pop-in or pop-out of the frame. Other proposed methods, such as space carving [16], would bypass some of the incremental building problems by performing what is essentially a subtractive reconstruction from multiple perspectives. However, these methods assume that you can accurately acquire the mask, which can be impossible in certain lighting conditions.

A majority of current algorithms for performing 3D object reconstruction have limitations: objects must be monitored from a large number of views; or views must follow a small baseline, thus the methods cannot function properly when provided only a small number or a single view. To solve these issues one of the most novel approaches employed for state-of-the-art reconstruction algorithms is to employ a priori knowledge of the objects that are being reconstructed [17,18]. These are generally relying on black-box models such as neural networks (NN). One of the most obvious advantages of using a priori information is for the algorithm to approximate the occluded object information, which we as humans are capable inferring quite easily. These methods have shown success in solving this task. For example, 3D Recurrent Reconstruction Neural Network (3D-R2N2) for multi-view reconstruction on the Sanford Online Products [19] and ShapeNet [20] datasets, has managed to achieve this task with fewer images available with competitive results [21], with the proposed improvement that uses densely connected structure as encoder and utilizing Chamfer Distance as loss function [22]. Additionally, Generative Adversarial Networks (GANs) can be used to generate 3D objects from multiple 2D views [23] or even from a single image [24]. GANs have also been shown to be able to predict former geometry of damaged objects [25]. Other authors have used feedforward NNs to detect valid matches between points in an image using different views with more than 98% accuracy [26]. Additionally it was shown that by adopting Bernstein Basis Function Networks (BBFNs) it is also possible to solve the task of reconstructing a 3D shape [27]. A trilateral convolutional neural network (Tri-CNN) that uses three dilated convolutions in 3D to extend the convolutional receptive field was applied on the ShapeNet and Big Data for Grasp Planning [28] data sets to obtain 3D reconstruction from a single depth image [29].

A majority of methods are using voxel based representations, e.g., PointOutNet [30] has shown the ability to predict and generate plausible 3D object shapes. This allows for the model to perform multiple predictions from a single input and using point cloud distribution modeling to refine the final results. Other approaches include: hierarchical surface predictions (HSPs) [31] for predicting high resolution voxel grids using convolutional neural networks (CNNs); discrete wavelet transform (DWT) and principal component analysis (PCA) can be used to get targeted object models, which can be used as an input to an artificial neural network (ANN) to recognize the 3D shape. Other authors have used geometric adversarial loss (GAL) in order to regularize single-view 3D object for object reconstruction using a global perspective by training the GAN to reconstruct multi-view valid 3D models [32]. RealPoint3D network composed of an encoder, a 2D-3D fusion module, and a decoder, accepts a single-object image and a nearest-shape retrieved from ShapeNet to generate fine-grained point clouds [33]. Similarly, PGNet [34], a recurrent generative network, uses the original images and partial projection images for fine-grained 3D reconstruction. Finally, it was shown that using ANNs it is possible to produce a fully textured, appropriately proportioned 3D model from a single RGB [35] or RGB-D frame [36], however, this approach was limited to basic volume primitives (rectangular boxes and spheres).

Even though the black-box methods have shown substantial improvements over existing state-of-art reconstruction algorithms such as incremental reconstruction, they can still be prone to severe mishaps due to poor illumination conditions, and object material interaction with light (mainly reflectivity). Furthermore, due to the fact that these methods rely on the visible light spectrum, they are incapable of working in dark environments. Therefore, they would not be suitable to be used in critical applications such as security.

Starting with the *Microsoft Kinect* released in 2010 [37] to *Intel Realsense* [38], the depth sensors are becoming the norm not only in the flagship mobile phones. As of late, stereoscopic depth is becoming available in newer budget phones with the introduction of multiple back facing cameras on a single device. For these reasons we have almost reached an era of the RGB-Depth (RGB-D) sensors being readily available. Therefore, focusing solely on the RGB cameras is missing the potential that the RGB-D cameras may provide for the object reconstruction tasks. For example, depth data stream from the Kinect camera has been used to generate topologically correct 3D mesh models [39].

Applying additional information provided by the RGB-D senor is the logical next step in the lifecycle of the object reconstruction algorithms as we believe they are less dependent on ambient conditions and could potentially be used in pitch black situations due to modern depth sensors using infrared cameras for object depth calculations on the hardware level. We concede that the depth sensors have their own limitations such as speckling due to surface properties [40,41] and distortions caused by infrared projections [42]. However, we believe that the addition of the depth sensor information in conjunction with readily available color data adds useful information. This information helps ANNs to better generalize input data and increase robustness against different lighting conditions. This includes pitch black environments as the depth information is sufficient to reconstruct the captured scene in most cases.

We present an improved hybrid ANN architecture for reconstructing polygonal meshes using only a single RGB-D frame, and employing a priori knowledge, which allows the neural network to be deployed on low-end RGB-D sensor devices with low frame rates.

## 2. Materials and Methods

### 2.1. Proposed Hybrid Neural Network Architecture

**Figure 1:**
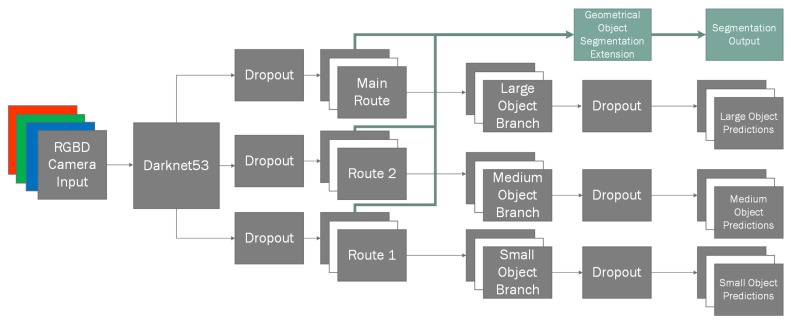
Our extended *YOLOv3* capable of extracting geometric object segmentation along with object bounding boxes.

Our hybrid NN architecture (Figure 1) consists of two major branches: the preliminary input branch that is used for object instance classification and their mask extraction; secondary input branch, which uses the results of preliminary branch in conjunction with the inputs of preliminary branch to perform individual object reconstruction. However, unlike preliminary branch we do not use generalized branches for reconstruction, instead we have *n* of specialized branches for each of the object categories. This allows us to more easily train additional types of objects in the reconstruction branches without having to re-train for classification, in addition this allows to re-train any of the individual reconstruction branches without losing the existing gradients by performing the training on more models [43]. The modularity of the system also provides the advantage of reduced training times as each branch can specialize onto its own generalization task, which gives the ability to change the network configurations of the reconstruction branches by simplifying for easier objects or having more elaborate ANN structures for more complex objects.

### 2.2. Classification and Segmentation Algorithm

Our aim is to detect individual object instances in the scene in order to have a system that is usable in real-world environments. Therefore, we need a classifier that is capable of detecting more than a single object instance for given frame, for example, having two cups and a toy plane on a table would require us to rebuild both of the cups and the toy plane models, respectively. Fortunately, some research has already been performed in the area of individual object instance classification [44,45,46].

For this reason, to perform our classification task we use one of existing state-of-the-art classifiers as it has shown to produce some of the best results in classification tasks, i.e. *YOLOv3* [47], which we have adapted to our needs to output an additional geometric segmentation mask (Figure 1), while authors have mentioned to be unable to achieve object instance segmentation in their original paper. Additionally, we define the term geometric segmentation as extension to segmentation that allows to discriminate between nearby object instances. This is done by generating a heatmap glow that radiates from the origin of the object. While other more lightweight methods exist, such as *MobileNet* [48], in our paper we try to compare the classification results using three different methods: using only color information; using only depth information; using both color and depth information. Therefore, we have decided to use a slower, but more accurate algorithm to have the most representative results.

Just as the majority of the individual object instance classifying algorithms, *YOLOv3* uses what is know as anchors for object detection. These anchors are used as jumping off bounding boxes when classifying objects, for example, a motor vehicle has a very different profile from a basketball. While the basketball in most cases has close to 1:1 aspect ratio bounding box, meaning that their width is the same, or very close when the image is distorted, to its height, while a motor vehicle like an automobile for the most part has height that is lower than its width. For this reason, one anchor could specialize in detecting automobiles, while the other can specialize in detecting basketballs. Additional feature, albeit a less useful one due to the way our training and testing dataset is generated, is the specification of bounding box scales by the authors of *YOLOv3*. These size specializations group bounding boxes into three groups: small, medium and large. For example small objects may include kitchen utensils, medium objects may include people, large objects may include vehicles. However, these bounding box groups are not exclusionary for these objects unlike anchors as these can vary a lot based on the camera distance from the object. Therefore, as our dataset is completely uniformly generating object scales this grouping loses some of its usefulness.

**Figure 2:**
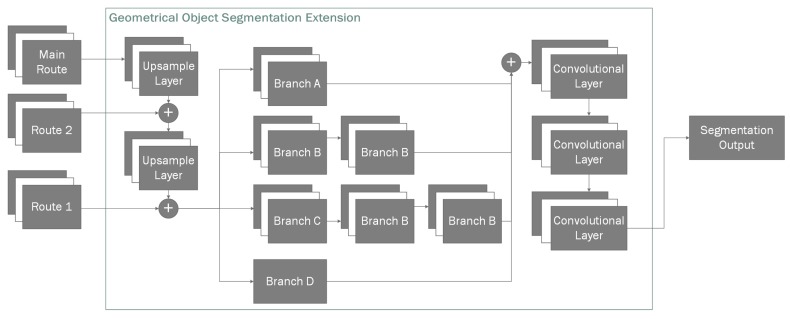
Our proposed geometrical object segmentation extension.

In our work, we have experimented with three types of inputs into the ANN: color space, front-to-back object depth field and the combination of both. In the case of color space, we use 3 channel inputs for representation of *red*, *green*, *blue* colors; when using depth field, we use a single channel input containing only normalized depth field values and for the combination of both we use *RGBD* channels in the same principle. Depth value normalization is performed by dividing each pixel *z* value using $z_{max}$ of the frame thus landing the depth in range of z=[0,1]. Our input layer is thereafter connected to *DarkNet53* network containing 53 convolutional layers as per specifications, which outputs three routes: *main route* used generally used for larger objects, *route 2* used for medium sized objects and, finally, *route 1* for smaller objects. Due to testing set being uniformly randomly generated, and containing the same object in potentially all size categories, we lose some of the flexibility that is provided by this setup and it impacts classification performance minimally, if removed. However, to stay true to the original algorithm and have an as unbiased result as possible, we have decided to keep all of the branches used in the source material. Additionally, these three routes provide good jumping off points for shortcuts to be used in our segmentation extension (Figure 2).

Due to each of the nearby routes being separated by the power-of-two scale, we use transposed convolutional layer [49] to upscale them gradually and then and merge them into desired final shape matrix. We construct our classless geometric segmentation mask by firstly upscaling the *main route* output and merging it with *route 2*, and the resulting layer is then upscaled again and merged with the final *DarkNet* output (*route 1*) which provides us a layer containing latent information of all previous layers that are each specified in learning different sized objects.

Next, we branch out our resulting hidden nodes into four different layers. Each layer contains slightly different network configuration, allowing them to essentially vote on their influence in the final result by extracting different latent feature-maps from the previous layers (Table 1). The first three branches (*A, B, C*) are convolutional branches containing one, two and three convolutional layers, respectively. However, for our final branch (*D*) instead of the convolutional layer, we use a max pool layer to extract the most prominent features. We have selected this parallel stacked approach, because we found it to be more efficient in extracting the object masks than linearly stacked layers when training the segmentation layers independently from the entirety of a model. This decoupling of the segmentation task from the classification task when training gives the additional benefit of allowing us to use transfer learning, which has shown to have very good practical results [50].

Next, we run our concatenated branches through convolutional layers to extract the most viable features and normalize their output in the range of (0, 1) giving us the final segmentation image. In our case the final segmentation output is 80 × 60 due to it being more than sufficient to extract approximate depth masks as we do not require pixel perfect segment representations. Finally, we use cascading flood-fill (Algorithm 1) to classify the masks pixels-wise. This is done because we found the generated binary masks to be impervious to false positives and false negatives, unlike classification using bounding boxes which can have three types of errors: false positives, false negatives and misclassification. This allows us to remove false positive bounding box detections when they do not intersect the origin of the mask. In our testing set, best cascade parameters were ϵ=0.9, θ=0.01.
**Algorithm 1** Cascading flood-fill1: **procedure**
GET_SEED(box,mask,ϵ)▹ Seeds initial values.2:  cx,cy←box▹ Get center for box.3:  seed← ∅4:  seed←find_closest_max(box,mask)▹ Find closest max pixel within bounds.5:  **if**
$seed\neq \emptyset \wedge seed_{value}\geq \epsilon$
**then**6:   $seed_{id}\leftarrow box_{id}$▹ Set seed id to box id7:   **return**
seed▹ Return seed if value greater than ϵ8:  **end if**9:  **return** ∅▹ No valid seed was found.10: **end procedure**11: **procedure**
FILL_NEIGHBOURS(seed,θ)▹ Recursively fill free neightbours with same or lower values.12:  **for each**
$n\in seed_{neightbours}$
**do**▹ For every neighboring mask pixel.13:   **if**
$n_{id}=\emptyset \wedge n_{value}\leq seed_{value}\wedge n_{value}>\theta$
**then**14:    $n_{id}\leftarrow seed_{id}$▹ Set neighbor to same id as seed.15:    FILL_NEIGHBOURS(n)▹ Call recursively.16:   **end if**17:  **end for**18: **end procedure**19: bounding_boxes←sort_confidence(bounding_boxes)▹ Sort bounding boxes by confidence.20: **for each**
box∈bounding_boxes
**do**▹ For each bounding box *b*21:  seed←GET_SEED(box,ϵ)22:  **if**
seed≠ ∅ **then**23:   FILL_NEIGHBOURS(seed,θ)24:  **end if**25: **end for**

Additionally, we have also modified *YOLOv3* network for we had issues with the network being unable to train by consistently falling into local minima during gradient descent and getting perpetually stuck in them. To solve this issue we introduced periodic hyper parameters [51] during model learning. Specifically, we had changed the learning rate to alternate in specified range of $lr_{min}=1e^{-6}$, $lr_{max}=1e^{-4}$.

(1) $$y\left(x\right)=\left\{\begin{array}{cc} \frac{x}{w_{1}}\times \left(lr_{max}-lr_{min}\right)+lr_{min}, & ifx<w_{0}\\ \frac{e^{1+\pi \times \frac{\cos\left(x-w_{1}\right)mod\left(w_{0}+1\right)}{w_{0}}}}{\pi e^{3}}\times \left(lr_{max}-lr_{min}\right)+lr_{min}, & otherwise \end{array}\right.$$

This periodical learning rate (Equation (1)) has vastly improved our models ability to learn the underlying relationships of input date by alternating between low and high training rates, therefore jumping out of potential local minima that it might start orbiting around during stochastic gradient descent. Our function has two stages, the first stage that consists of two training iterations, where $w_{1}=2\times s$, and the second stage of 4 iterations, where $w_{0}=4\times s$ where *s* is the number of steps per batch. We selected the two state learning function because having high learning rates initially may cause the model to diverge. Therefore, during the first stage we linearly increase the learning rate. Once in the second stage we use the cosine function and the modulus operator for the model to alternate between two values. The shape of the alternating function also can have influence in model convergence as some models require to be in different extremum points for different amounts of times. Therefore, having a different dataset may require more fine-tuning of parameters of this equation for different slope shapes, while still maintaining the benefits of having alternating learning rates.

Additionally, as we are training the NN from scratch, we have noticed that our network, despite being able to find better convergence results due to periodical learning rate jumping out of local minima, had a high bias rate. A high bias rate is an indicator that our model is over-fitting on our data set. To solve this additional issue, we modified the *YOLOv3* network by adding additional dropout layers with the dropout rate of P(x)=0.5 after each branch of *DarkNet53* and before each of the final layers predicting the bounding boxes.

Furthermore, we had issues of model overfitting to the training set, to solve this we additionally modified the neural network by adding two additional dropout layers. We trained our model 6 times, each with 50 iterations using mini-batch of size 8 for comparison, because after about 50 iterations the standard *YOLOv3* model starts to overfit and loose precision with our validation dataset. Therefore, for most objective comparison we trained our modified network for same number of epochs. Note that even though our method also starts to overfit, unlike the YOLOv3 network model, the accuracy of our modified model when overfitting remains roughly at the same value from which we can deduce that the changes make the model more stable.

**Figure 3:**
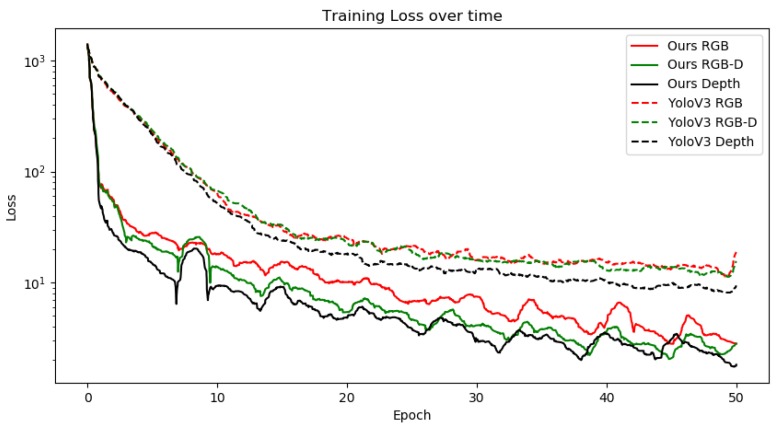
Training loss comparison between baseline *YOLOv3* and our modified version when using RGB, RGB-D and depth data as training. Due to the loss function being inherently noisy for each of the mini-batches, we have used Savitzky-Golay [52] digital filter to perform the smoothing of the overall graph.

Figure 3 shows the differences in loss function when trained using the RGB, RGB-D and Depth data as input. For the unmodified *YOLOv3* we are using $lr=1e^{-5}$ as the midpoint between our minimum and maximum learning rates in the periodic learning rate function. As we can see from the graph, the loss function using static learning rate on the RGB and RGB-D datasets reaches a local minimum causing the model to slow down its ability to learn new features, unlike our periodic learning rate which seems to temporarily force the model to overshoot its target which sometimes causes it to fall into a better local minimum. This effect can be seen in the distinct peaks and valleys in the graphs. The outlier in these graphs are depth-only data points. While in both cases the loss function seems lower and has a better downwards trajectory in stochastic descent, however, we have noticed that despite seemingly lower loss when compared to RGB and RGB-D, the actual model accuracy is very unstable on epoch-per-epoch basis. We assert that this is the case due to depth alone providing very unstable data that’s very hard to interpret. We make this assumption due to the fact that even when taken an expert to evaluate the depth maps alone, it is usually very hard to discern what type of object it is without knowing its texture; it is only possible to tell that there is in fact an object in the frame. Finally, we can see that the RGB-D data is a clear winner when training in both cases, which means that depth data can indeed help in model generalization.

### 2.3. Reconstruction Algorithm

**Figure 4:**
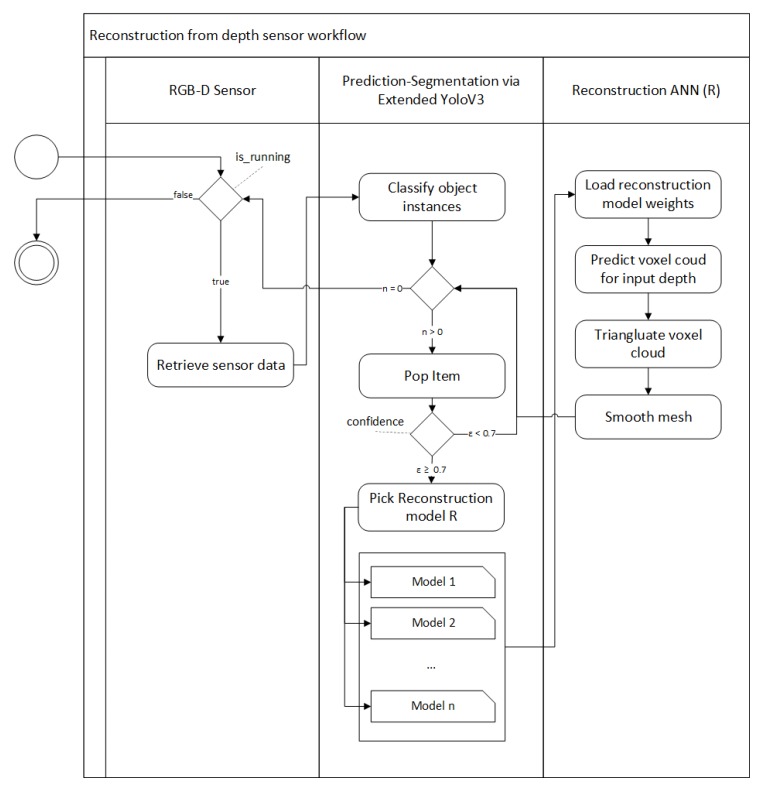
Workflow of object reconstruction from sensor data.

**Figure 5:**
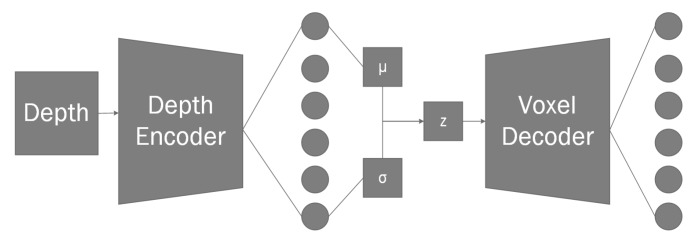
Diagram of a single object reconstruction network architecture branch. For the given depth frame, the depth encoder creates a bottleneck, which is then directly connected to VAE node, the resulting sampler is connected into voxel decoder. The voxel decoder layer outputs a 32×32×32×2 matrix which can be explained as x×y×z×s, where *x, y, z* components indicate position in 3D grid, and *s* component indicates voxel state encoded as one-hot.

The proposed algorithm for 3D object reconstruction consists of two subsystems: voxel cloud reconstruction and post-processing (Figure 4). In reconstruction step we take the outputs of the 3D classifier mask for the object and in conjunction with the original depth map which we feed into our reconstruction ANN (Figure 5) that performs the object reconstruction task for the given masked input frame. Unlike the classification algorithm we only use the underlying depth input from the classifier as it provides enough information for the specific object reconstruction. This is due to fact that we already know the class of the object, which is required for classification because different objects can have very similar depth representations. However, during reconstruction this is not an issue because our ANN is designed in such a way that each branch is responsible for reconstructing similar object representations.

Once the classifier-segmentation branch has finished its task, for each object instance the appropriately trained reconstruction branch is selected. In our case all the branches are highly specialized on a single type of object that it can reconstruct, which is why object classification is required. However, we believe that there is no roadblock to having more generic object reconstruction branches for example all similar objects may be grouped to a single reconstruction task. This could potentially allow some simplifications in the classification-segmentation as it would no longer be required to classify highly specific object instances thus reducing failure rate caused by object similarities. For example, a cup and a basket can be very similar objects and be misclassified. Additionally, the hybridization allows for fine tuning of the reconstruction branches without having to retrain the entire neural network model potentially losing already existing gradients via on-line training skewing the results towards new data posed. This in turn reduces re-training time if new data points are provided for a specific object as we no longer need to touch the established branches due to modularity.

**Figure 6:**
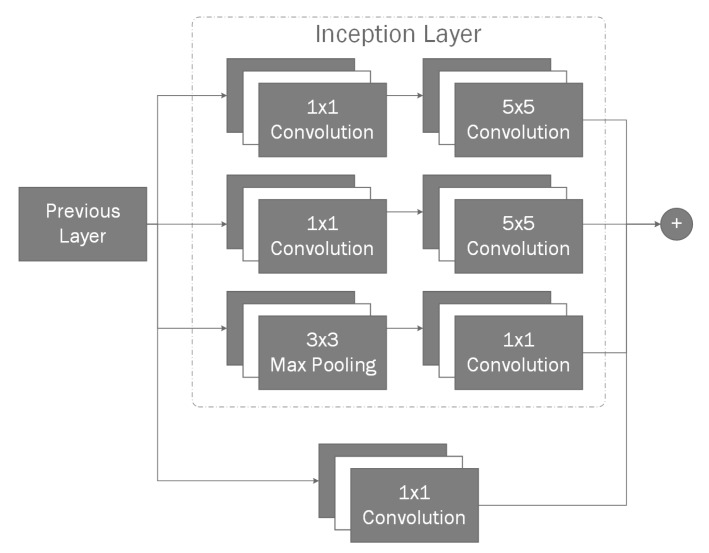
An example of the inception layer. An input layer is connected to three branches in parallel. If multiple inception layers are used inception layers are connected sequentially. Final inception layer outputs and 1 × 1 convolution are then connected using addition. The result is then used for subsequent layers.

Inside our reconstruction network branch (Figure 2) for given depth input we use convolutional layers to reduce the dimensionality of the input image during the encoding phase (see Table 2). For a given input, we create a bottleneck convolution layer which extracts 96 features, afterwards we use a spatial 2D dropout [53] layer before each with P(x)=0.1 to improve generalization. We use spatial dropout as it is shown to improve generalization during training as it reduces the effect of nearby pixels being strongly correlated within the feature maps. Afterwards, we add an additional inception [54] layer (Figure 6) which we will use as a residual block [55] followed by another spatial dropout. Afterwards, we add two additional bottleneck residual layers, each followed by additional dropouts. With final convolution giving us final 256 features with the resolution of 20 × 15. Our final encoder layer is connected using a fully-connected layer to a variational autoencoder [56] containing 2 latent dimensions, as variational autoencoders have shown great capabilities in generative tasks. Finally, the sampling layer is connected to full-connected layer which is then unpacked into a 4 × 4 × 4 matrix. We use the transposed three-dimensional convolutional layers in order to perform up-sampling. This is done twice, giving us 4 feature maps in 32 × 32 × 32 voxel space. Up to this point we have used Linear Rectified Units [57] (ReLUs) for our activation function, however, for our final 3D convolutional layer we use a softmax function in order to normalize its outputs where each voxel contains two neurons. One neuron indicating the confidence of it being toggled on, the other neuron showing the confidence of the neuron being off. This switches the task from a regression task to a classification task, allowing us to use categorical cross entropy to measure the loss between the predicted value and our ground truth.

### 2.4. Proposed Network vs. YOLOv3

**Figure 7:**
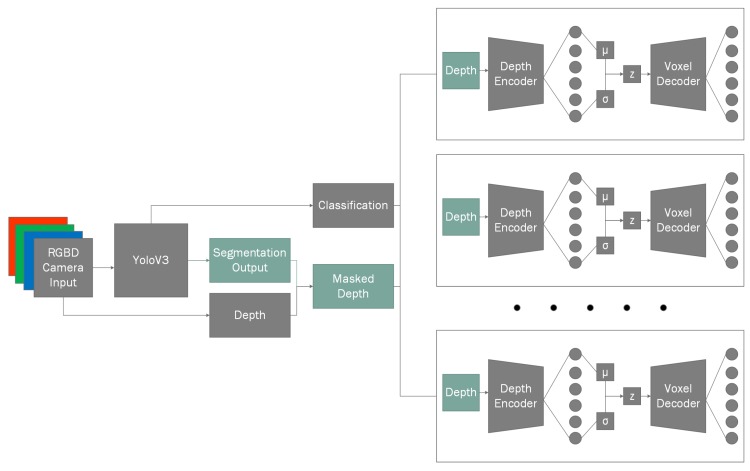
Full view of the proposed network model that extends the YOLOv3 network.

Our approach is the hybridization of two ANN architectures: classification-segmentation branch and reconstruction branch (see Figure 7). The classification-segmentation branch as the name suggests performs object instance classification and segmentation. This information is then fed to the object reconstruction branches. Object reconstruction branch contains a fleet of specialized pre-trained autoencoder models where each of the auto-encoders can reconstruct the model’s three-dimensional representation while being provided only a single depth frame. The initial classification-segmentation branch is our expanded interpretation of YOLOv3 which adds crucial output to already existing YOLOv3 network output, i.e., the object instance segmentation. This extension adds crucial information which is required for the reconstruction step by extracting the object instance mask that can be applied per each object on the initially captured depth.

### 2.5. Dataset

**Figure 8:**
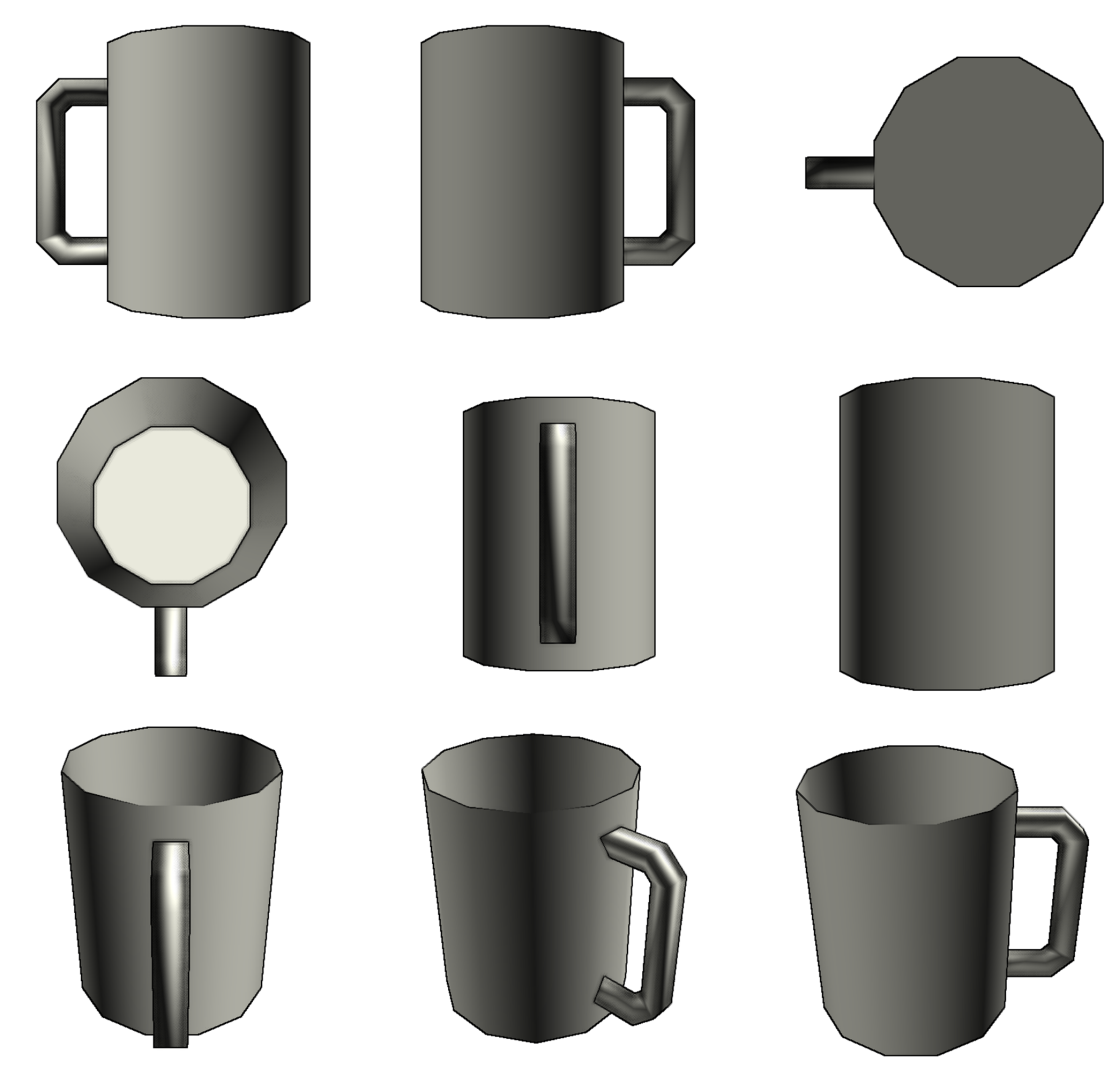
A coffee cup model from the *ShapeNetCore* dataset.

As our method entails the requirement of a priori information for the captured object reconstruction, there is a need for a large well labeled element dataset. However, unlike for object recognition which has multiple datasets, e.g., *COCO* [58] dataset, *Pascal VOC* [59]; there seems to be a lack of any public datasets that provide RGB-D scene representation in addition to it’s fully scanned point cloud information viable for our approach. While datasets like *ScanNet* [60] exist, they are missing finer object details due to focusing their scan on full room experience that we are trying to preserve. Therefore, our training data consists exclusively out of synthetically generated datasets, which use the *ShapeNetCore*, a subset of *ShapeNet* dataset that provides 3D object models spanning 55 categories (see an example of a coffee cup model in Figure 8). In addition, we use real-life data acquired by the *Intel Realsense ZR300* and *Intel Realsense D435i* (Intel Corp., Santa Clara, CA, USA) devices for visual validation as it is impossible to measure it objectively without having a 3D artist recreating a 1:1 replica of said objects, which is unfortunately unfeasible option. However, using real world samples as a validation set is not subject to training bias because they are never being use in the training process.

As mentioned, for the training of the black-box model we are using the *ShapeNetCore* dataset that we prepare using *Blender* [61] in order to create the appropriate datasets. Due to the fact that we are training a hybrid neural network, we need two separate training and testing sets, one for each task.

#### 2.5.1. Classification Dataset

To create this subset of data we create random scenes by performing the following procedure. Firstly, we randomly decide how many objects we want to have in the scene in the range of $n_{objects}=\left[1;10\right)$ and pick that many random objects from *ShapeNetCore* dataset to populate the scene. Before applying any external transformations we transform the object geometry so that all objects are of uniform scale and have the same pivot point. To perform the required transformations firstly we calculate the geometry extents. Once we know the object extents we can move all the objects on *Up* axis (in our case this is *z*) and scale down all vertices by the largest axis (Algorithm 2). This gives us a uniformly scaled normalized geometry that we can freely use.
**Algorithm 2** Normalize geometry1: **procedure**
Extents(*G*)▹ Calculates extents for geometry *G*2:  $min_{x},min_{y},min_{z}\leftarrow Infinity$▹ Initialize min vector3:  $max_{x},max_{y},max_{z}\leftarrow -Infinity$▹ Initialize max vector4:  **for each**
v∈G
**do**▹ For each vertex *v*5:   $min_{x}\leftarrow min\left(v_{x},min_{x}\right)$6:   $min_{y}\leftarrow min\left(v_{y},min_{y}\right)$7:   $min_{z}\leftarrow min\left(v_{z},min_{z}\right)$8:   $max_{x}\leftarrow max\left(v_{x},max_{x}\right)$9:   $max_{y}\leftarrow max\left(v_{y},max_{y}\right)$10:   $max_{z}\leftarrow max\left(v_{z},max_{z}\right)$11:  **end for**12:  **return**
min,max13: **end procedure**14: min,max←EXTENTS(G)15: bounds←max−min16: $max\_bound\leftarrow 1/max(bounds_{x},bounds_{y},bounds_{z})$17: **for each**
v∈G
**do**▹ For each vertex *v*18:  $v_{x}\leftarrow v_{x}/max\_bound$19:  $v_{y}\leftarrow v_{y}/max\_bound$20:  $v_{z}\leftarrow \left(v_{z}-min_{z}\right)/max\_bound$▹ Offset the vertex on *up* axis before normalizing bounds21: **end for**

We place the selected objects with random transformation matrices in the scene, making sure sure that the objects would never overlap in space. To generate random local transformation matrix (*L*) (Equation (3)) we need three of it’s components: Scale (*S*), Rotation ($R_{z}$) and with random value; use either capital or lower-case s in both places in the range of s=[0.7,2); Rotation ($R_{z}$), where rotation is random value in the range of θ=[0,2π), we perform rotation only on *z* axis to ensure that randomly generated scenes are realistic and do not require artist intervention; Translation (*T*), where *x* and *y* values are non-intersecting values in the range of r=[−5,5] and α=[0,2π) (Equation (2)).

(2) $$\left\{\begin{array}{c} x=r\times \cos\alpha \\ y=r\times \sin\alpha \end{array}\right.$$

(3) $$L=S\times R\times T=\left[\begin{array}{cccc} s & 0 & 0 & 0\\ 0 & s & 0 & 0\\ 0 & 0 & s & 0\\ 0 & 0 & 0 & 1 \end{array}\right]\times \left[\begin{array}{cccc} \cos\theta & -\sin\theta & 0 & 0\\ \sin\theta & \cos\theta & 0 & 0\\ 0 & 0 & 0 & 0\\ 0 & 0 & 0 & 1 \end{array}\right]\times \left[\begin{array}{cccc} 0 & 0 & 0 & 0\\ 0 & 0 & 0 & 0\\ 0 & 0 & 0 & 0\\ x & y & z & 1 \end{array}\right]$$

Once the selection objects are placed we need to apply lighting in order to have real-life like environments. To do this, we use the Lambertian shading model and directional lights. We randomly generate $n_{lights}=\left[1;4\right)$ lights in the scene. We pick a random light rotation, we ignore translation as it does not matter in directional lights; we generate a random color in the range of $Col_{RGB}=\left[0.7,1\right]$, we selected the minimum bound of 0.7 to avoid unrealistic real-world lightning; and random intensity I=[0.7,1]. This light acts as our key light. To avoid hard shadows being created, which wouldn’t be the case unless using spotlight in real world, for each key light we create a backlight which is pointing the opposite direction of key light with half the intensity and identical color to the key light.

Once the scene setup is complete, we render the scene in three modes: *color*, *depth* and *mask*. Color mode gives us the scene representation from a regular light spectrum camera. As we are not putting any background objects into the scene the generated background is black. However, later on we use augmentation during training to switch the backgrounds to improve recall rates. Once the color frame is extracted we extract the mask, in order to extract the mask we assign each object an incremental *ID* starting at 1, this allows us to differentiate between objects in the frame. Finally, we render the depth representation of the scene. Before rendering depth we place a plane on the ground that acts as our ground place, this allows for more realistic depth representations because the objects are no longer *floating* in space. The depth is rendered front-to-back, meaning the closer the object is to the camera the closer to zero depth value is, the front-to-back model was chosen because this is the same as *Intel Realsense* model.

Each of the scenes is rendered in *320 × 240* resolution n=25 times by placing it in random locations (Algorithm 3) and pointing it at the center of the scene, where r=10, $z_{min}=4$, $z_{max}=6$.
**Algorithm 3** Camera location1:step_size←2π/(1−n)2:**for** i < n **do**3:  θ←random(i,i+1)        ▹ Random float in the range of [i, i+1]4:  x←cos(step_size×θ)×r5:  y←sin(step_size×θ)×r6:  $z\leftarrow random(z_{min},z_{max})$7:**end for**

We save the perspectives as *OpenEXR* format [62] instead of traditional image formats instead of, for example, *PNG*, as *OpenEXR* file format is linear, allowing for retention of all depth range without any loss of information as it is not limited to 32 bits per pixel. The final *EXR* file has these channels in it *R*, *G*, *B* containing red, green and blue color information respectively; *id* channel contains the information about the mask for specific pixel; *Z* information containing the linear depth data.

Once we create the input image, we additionally label the data and extract the segmentation mask that will be used as output when training the artificial neural net. We perform this step after the scene is rendered in order to account for any kind of occlusion that may occur when objects are in front of each other causing them to overlap. We extract the object bounding boxes by finding the most top-left and bottom-right pixel of the mask. The binary mask is extracted based on the pixel square distance from the center of the bounding box. This means that the center pixels for the bounding box are completely white and the closer to the edges it is the darker it gets. We use non-flat segmentation to be able to extrapolate individual object instances in the mask when they overlap, and this is done by interpolating the pixel intensity from the bounding box edge to bounding box center. The mask is then scaled down to *80 × 60* resolution as it is generally sufficient and reduces the required resources.

#### 2.5.2. Anchor Selection

**Figure 9:**
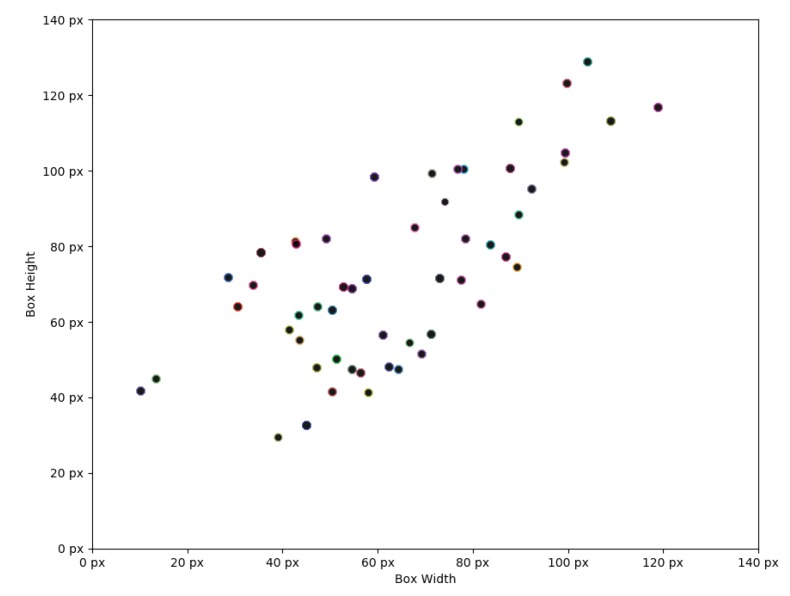
Each individual point denotes the mean object bounding box scale for each class type.

The existing anchors that are being used with *COCO*, *Pascal VOC* and other datasets are not suitable for our dataset, rarely fitting into them. Therefore, we performed class data analysis and selected three most fitting anchors per classifier branch scale. As we can see from Figure 9, our classes generally tend to be biased towards 1:1 aspect ratio due to data set being randomly generated unlike in real world applications.

**Figure 10:**
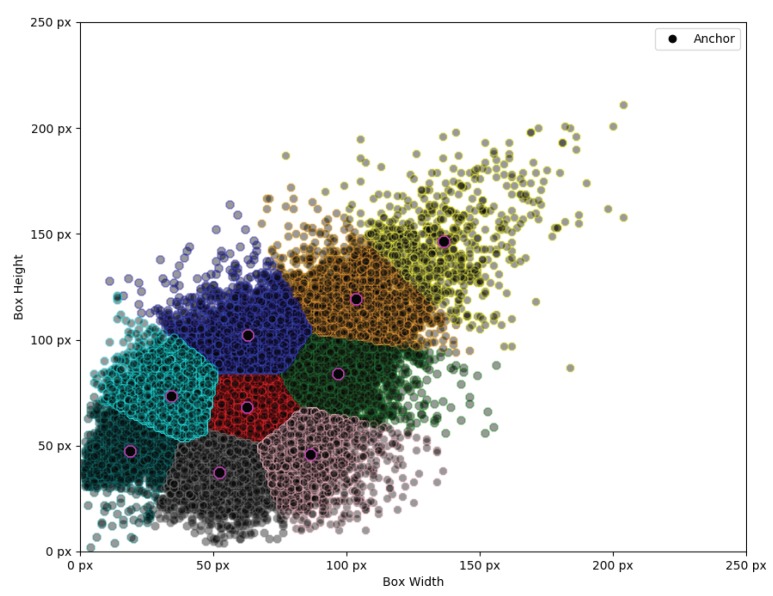
Selected anchors using *K-Means* clustering algorithm. Different colors denote distinct anchor groups responsible for detecting objects in the spread.

However, while the classes tend to be biased towards 1:1 for the most part, the assertion that all individual object instances would neatly fit into this aspect ratio would be incorrect as they still retain certain bias. According to previous Single Shot Detection (SSD) research [63], selecting inadequate base anchor boxes can negatively affect the training process and cause the network to overfit. Therefore, we chose to have 3 anchors per anchor size as this seems to sufficiently cover the entire bounding box scale spread by including tall, wide and rectangle objects. We select the anchor points using *K-Means* to split data into 9 distinct groups (Figure 10).

Once we have our cluster points for bounding box detections, we sort them in order to group into small, medium and large anchor sets. Giving us three different anchors, each having the most popular aspect ratios per that scale detection branch as it can be seen in Table 3.

The neural network architecture described in Section 2.2 was trained in three separate modes in order to infer how much the additional depth information improves the classification results. These three modes consist of RGB, RGB-D and Depth training modes. Where RGB mode implies we train using only the color information that was generated from the dataset, the RGB-D mode uses both depth and color information and finally Depth mode trains the network using only depth information. We do not use any additional data augmentation when training in both RGB and RGB-D modes. We do however, add additional augmentation when training in the RGB-D mode. When training in the RGB-D mode there is a small chance that either RGB or Depth channel will not be included in the testing sample. We perform this augmentation because both RGB camera and Depth sensors may potentially have invalid frames. Therefore, we assert that both of these data points are equally necessary for the classification task, and that they must be generalized separately from each other and should provide equal contributions to the classification task. This is decided randomly when preparing the mini-batch to be sent to the artificial neural network for training. There is λ=0.1 chance that the input specific data point will be picked for additional augmentation. If the data point is picked for augmentation then there is equal probability that either RGB or Depth Data will be erased from the input and replaced with zeros. We decided on this augmentation approach because both RGB and Depth frames using real sensors are prone to errors. For example, the RGB camera may fail in bad lighting or even be unavailable when the room is pitch black. Likewise, the depth frames are also prone to errors due to inconsistencies in generating depth map which causes the sensor to create speckling effect in the depth information, additionally cameras being too close to object may be completely unable to extract proper depth information. Therefore, we chose this augmentation approach as it allows for the sensors to work in tandem when both are available, but fill in the gaps, when one of them is failing to provide an accurate information.

#### 2.5.3. Reconstruction Dataset

For the reconstruction training set, we use the same *ShapeNetCore* dataset to generate the corresponding depth images and ground truths for the individual objects voxel cloud. We used *Blender* to generate the training data. However, the generated input data is different. We assert that the object material does not influence the objects shape, therefore we no longer generate the color map unlike when generating classification data. Therefore, we only render the depth information for each object. We render individual objects by placing the cameras in such a way that the specific object would be visible from all angles from 45° to 90° at a distance from 1 to 1.5 m, excluding the bottom. As a result we have 48 perspectives for each of the object models. Once again we save the models as *OpenEXR* file in order to preserve the depth values in this lossless format. Finally, we generate the voxel-cloud representation [64]. Voxelization is performed by partitioning into the equally sized cells, where the cell size is selected based on the largest object dimension axis. Following the space partitioning, we repeat over each of the cells and compute whether the specific cell should be filled by ray-polygon intersection [65].

### 2.6. Evaluation

In order to evaluate the correctness of our results, we evaluate the results of the proposed algorithm, and additionally we evaluate both of the subsystems individually. To evaluate the classification accuracy, we use the *mAP* metrics to assess the quality of the classifier and it’s output bounding boxes. When performing the classification accuracy evaluation, we evaluate all three train models: RGB, RGB-D and Depth. This allows us to determine the quality differences between the addition of depth information in the classification task.

For the reconstruction task we require the output voxel representation of the object to be as close to ground truth as possible. For that, we define our reconstruction quality as the *Intersection-over-Union* metric. Furthermore, we use the *Correctness*, *Completeness*, and *Quality* metrics during evaluation.

## 3. Results

### 3.1. Settings

Our experiments have been executed using two computers: (1) a workstation with *Intel i7-4790* CPU with *16 GB of RAM* which achieved 55.76 fps, and *nVidia 1070* graphics card with *8 GB GDDR5 VRAM*; and (2) a laptop computer using *nVidia 960M* graphics chip with *4GB GDDR5 VRAM*, *Intel i5-4210H* CPU and *12 GB of RAM*, which reached 11.503 fps. We consider that these machines should represent the target range of end user devices.

### 3.2. Quantitative Results

#### 3.2.1. Object Instance Classification Results

In order to evaluate our model in all cases, we have used the mAP metric, which is a widely used method in order to evaluate mean average precision of the predicted bounding boxes with respect to their Intersection-over-Union (IoU), provided that the object classes match. As per suggested *COCO* evaluation we filter out bounding boxes which have an IoU<0.5 in order to compare all of our trained model versions.

As Table 4 suggests, our iterative training approach in addition to dropout layer was substantially better in the object classification task as opposed to the originally suggested variant which would either plateau with too low of a learning rate or get stuck in a constant loop around the local minima due to the initial learning rate being too high. Therefore, we can assert that a periodic learning rate is a useful tool to improve model generalization and the speed at which the network can train by adding additional noise during training time in a form of sudden overshooting. Furthermore, we can see that the addition of depth information as input greatly increases the recall rate in both cases, while the depth information alone has similar recall rate in both cases. This suggests that the depth cameras can not only greatly benefit in the object classification task when used in conjunction with visible light spectrum cameras but it can be used as a fallback when no light source is available, albeit with lower precision.

**Figure 11:**
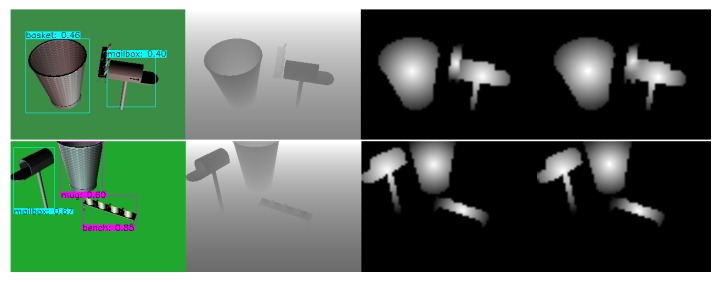
Prediction made with our extended *YOLOv3* network. Left to right: (1) Input color image with predicted object instances; (2) Input depth frame; (3) Upscaled to 320 × 240 ground truth mask; (4) Predicted mask upscaled to 320 × 240. Same object is being treated as two distinct classes due to lack of cues for the artificial neural network of what the specific object may be due to scenes being generated randomly.

One of the glaring issues we noticed with the *ShapeNetCore* dataset during our experiments is that, while there are specified a total of 55 classes, a lot of those classes have major overlap in form and function which may dramatically affect the overall mAP value, such as classes that are categorized as distinct (e.g., *pistol* and *handgun*) could still be grouped into the same class as they share key characteristics which may not be viable to differentiate when using relatively low resolution images. Additionally, some groups of objects can be distinct in their use (e.g., *mug* and *ashcan* and *basket*) in many cases have no differentiable features and would require each individual scene to be hand crafted by an artist in order to provide visual queues about the objects in relation to the world, which should potentially allow for differentiation between very similar objects (Figure 11). However, this is currently beyond the scope of our paper.

#### 3.2.2. Mask Prediction Results

As one of our main goals is to extract individual object instances from the depth map, we extended the *YOLOv3* network architecture to be able to predict object masks. In order to compare the predicted mask similarity with the ground truth we use the structural similarity index metric (SSIM) that measures perceived similarity between two images.

**Figure 12:**
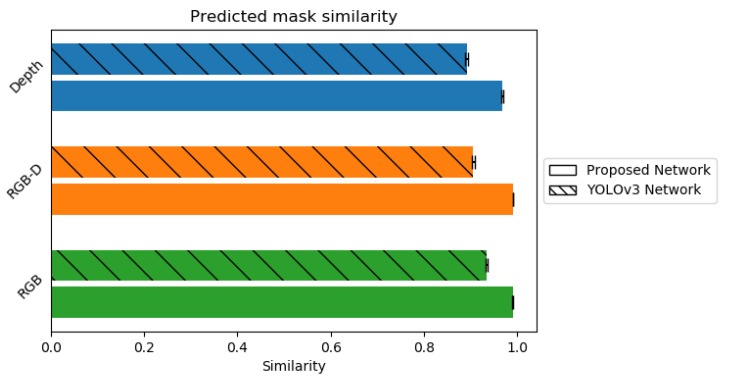
Similarity of created mask to the mask of ground truth. The hashed bar denotes the similarity of masks predicted by the *YOLOv3* network, the solid bar denotes the similarity of masks predicted for Depth, RGB-D and RGB frames by the network model proposed in this paper.

**Figure 13:**
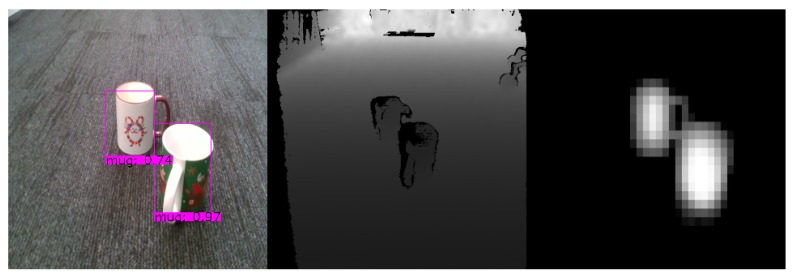
An example of real world object classification using the proposed network model: Segmented and classified RGB frame (**left**), depth frame (**middle**), and predicted depth mask (**right**).

As we can see from Figure 12, in all cases our *YOLOv3* extension for object mask prediction is capable of extracting mask frame not only from the combined RGB-D frames but also from the RGB and Depth frames alone. This shows us that both color and depth information individually is generally enough for this task. However, both of these sensors may fail in different environments so the conjunction of both would most likely procure the most accurate results. Additionally, while in both method cases (static and periodical) the similarity is generally more than enough to extract accurate mask, using periodical approach provides a much lower standard deviation, hence better expected results. Additionally, the higher similarity also signals a tighter mask which may improve reconstruction quality due to reduction in bad data. While in our tests RGB has slight advantage over RGB-D when generating a mask, it is worth noting that Depth adds an additional dimension to the data which makes the dataset slightly harder when compared to RGB alone. This is due to RGB alone being able to drop the randomly generated background, unlike RGB-D which has a non-uniform background due to addition of ground plane. As we can see in Figure 13, our approach is applicable not only for synthetic but for real-world data too. This indicates that the network managed to generalize well and it’s result can be used during reconstruction step.

### 3.3. Reconstruction Results

#### 3.3.1. Quantitative Results

**Figure 14:**
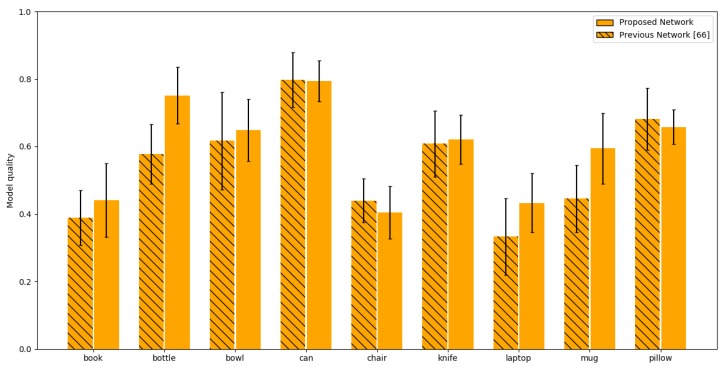
Comparison between the predicted object shape and ground truth using the IoU metric for different objects in the training set. The hashed bars denote the results achieved using the network proposed in [66]. The solid bars denote the results for the proposed network.

We can observe the achieved results for our proposed method in Figure 14 as they compare to previously achieved results in hybrid neural-network reconstruction [66]. As we can see the mean *IoU* metric value as compared to the results presented in [66] has significantly improved for some of the models, more importantly—even if the the improvement was minimal or if the results were slightly lower the error spread is lower. This indicates that the achieved results are much more stable. Additionally we can see that our reconstruction results are comparable to that of other state-of-art methods like *3D-R2N2* reporting 0.571 mean *IoU*.

#### 3.3.2. Visual Inspection and Result Validation

For every object that we have trained, we had collected real world examples using *Intel Realsense* device in order to compare how well synthetic results transfer into real world data. The results for the given dataset can be seen in the Table 5.

The reconstructed object shapes are generally recognizable. However, certain object angles cause the network to fail the task, for example, one of the bowls is missing half of it’s voxels, while the other bowl may be considered a near perfect reconstruction. While the ANN has managed to reconstruct the *Book* and *Knife* datasets, it has generally only managed to reconstruct their base shape primitives which make the objects somewhat indistinguishable by experts without any prior information of what the objects may be. While the human bias may notice the minute structural differences between the knife handle and blade in terms of it’s width, we still consider this a failed reconstruction. *Can* has managed to achieve great results in terms of reconstruction, while the pillow reconstruction could be considered near perfect. *Mug* in our training set is one of the trickiest objects as it contains a handle which should be reconstructed with a hole and additionally the mug cannot be fully filled in with voxels as in our case it is empty. While in all three cases the basic shape of the cup was maintained, there are some issues with two test cases. One of the test cases was missing a hole for the handle, while another is substantially distorted. However, the distortions may be explained by extremely noisy dataset. The *Chair* dataset allowed to reconstruct the shape of the chair although some of the details were missing. The *Laptop* and *Bottle* datasets are the hardest ones in terms of depth sensor capabilities. Depth sensor has issues in retrieving depth information for IR reflective surfaces causing it to distort the images fully. Such surfaces in our case are computer screen and a plastic bottle. However, the *laptop* data has surprisingly managed to account for this error in depth map, albeit containing some distortions.

#### 3.3.3. Reconstruction of Multiple Objects

**Figure 15:**
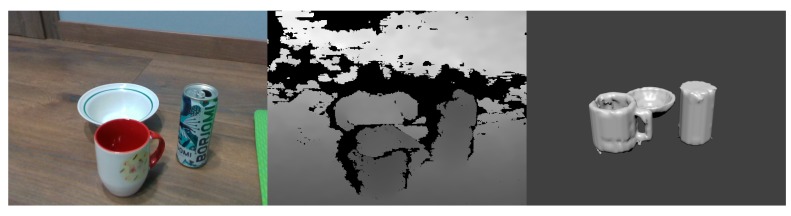
An example of reconstruction of multiple objects in the scene: Segmented and classified RGB frame (**left**), depth frame (**middle**), and predicted depth mask (**right**).

As a proof of concept, we have performed the experiments to reconstruct multiple objects in the scene (see an example of results in Figure 15). By extracting the individual object masks and performing an object reconstruction individually we have managed to reconstruct the specific objects in the scene. However, we are unable to predict the object’s relative position, rotation and scale in relation to camera space. For this reason, we have had to specify the object transformation in relation to camera and other scene objects manually to perform final scene render.

## 4. Discussion and Concluding Remarks

### 4.1. Discussion

One of the main advantages of our suggested hybrid NN based method is that unlike other non-hybrid approaches, it is relatively easy to include additional objects into the dataset, due to the fact that you can train network branches separately. Unlike other approaches, we do not need to re-train the model with all the previous data as we do not risk losing any of the existing gradients due to network being skewed to the new data points. The modularity of the approach allows us to train the network reconstruction nodes per each object category independently. Additionally, this modularity allows for variance of the model per object class, meaning we can adjust complexity of the ANN depending on the difficulty of the object that is being reconstructed. Furthermore, we believe that our approach is a step forward to generic object reconstruction as we are capable of extracting multiple objects from the same scene thanks to masking during classification step, which allows to send only the relevant objects depth information into the reconstruction node.

While our approach is capable of extracting the individual object instances and reconstructing them, additional research is required for full scene reconstruction. This feat requires finding the camera space matrices, paving the way for application in Extended Reality systems. One of the standing issues with our current approach in terms of reconstruction is that our ground-truths are perspective-invariant. This makes training the network slightly harder, additionally it may somewhat reduce the quality of the results due to network somewhat trying to adjust to observation angle, therefore making the IoU metric values lower, despite visually being feasible. Solving the perspective invariance may also be a partial solution to the homography [67,68] problem as our reconstructed object would already be rotated with respect to the camera space.

Additionally, the improvements on the dataset may be obtained by creating and incorporating a real-world dataset along with synthetic data for the depth encoding step. Thus, we can potentially improved results when using real depth sensors. Additional improvements to the network architecture may also be found by changing the complexity of the model [69]; pruning dead neurons [70]; using neuro-evolutionary and neuro-genetic algorithms to find a much more fitting solution [71]; enhancing the learning of the artificial neural networks by using metaheuristic control mechanism [72]; or using multiple frames from a video feed instead of the current single frame solution as a large number of depth frames from a single view may reveal some hidden features and improve recall rate [73]. Using multiple frames would allow for exploration of what improvements may be achieved with the use of recurrent neural networks (RNN) for they have shown to be capable of predicting sequential data [74,75,76]. Finally, using the RGB frames combined with depth frames for reconstruction can potentially add some missing features from the depth due to inherent noisiness of the sensor, therefore improving the recall rate [77,78].

Finally, we have compared the complexity of the proposed network model with the YOLOv3 network as well as with other popular network architectures. The results presented in Table 6 how that the proposed network model is only sightly more complex than YOLOv3 in terms of the number of model parameters and operation, but outperforms other network architectures in terms of operations.

### 4.2. Concluding Remarks

Our proposed hybrid artificial neural network modifications have allowed to improve the reconstruction results with respect to theYOLOv3 network results by 8.53% which allows for much more precise filling of occluded object sides and the reduction of noise during the process. Additionally, the reconstruction results are a lot more stable when compared to previous results. Furthermore, the addition of object segmentation masks and the individual object instance classification is a leap forward towards a general purpose scene reconstruction as opposed to single object reconstruction task due to the ability to mask out overlapping object instances and use only masked object area in the reconstruction process. While further research is needed in order to retrieve object orientation and position with respect to camera space, we believe our method allows for a much broader application in comparison to previous research due to its focus on single object reconstruction. 

## Figures and Tables

**Table 1:** Geometric Segmentation architecture.

	Type	Filters	Size	Output
Main route	Transposed Convolution	1024	2×2×2	20×20
	Concatenate	-	-	20×20
	Convolution	256	1×1	20×20
Route 2	Transposed Convolution	256	2×2×2	40×40
	Concatenate	-	-	40×40
	Convolution	256	1×1	40×40
	Upscale	-	-	160×120
Branch A	Convolution	128	1×1/2	80×60
Branch B	Convolution	32	1×1	160×120
	Convolution	128	1×1/2	80×60
Branch C	Convolution	32	1×1	160×120
	Convolution	128	2×2	160×120
	Convolution	256	3×3/2	80×60
Branch D	Max Pool	256	3×3/2	80×60
	Concatenate	-	-	80×60
	Convolution	256	1×1	80×60
	Convolution	128	1×1	80×60
	Convolution	1	1×1	80×60
	Clip Values	-	-	80×60

**Table 2:** Architecture of the reconstruction neural network.

	Type	Filters	Size	Output
	Input	-	-	320×240
Encoder	Convolution	96	5×5/2	160×120
	Dropout 2D P(x)=0.1	-		160×120
	Inception	(8, 4)	-	160×120
	Convolution	16	1×1	160×120
	Add	-	-	160×120
	Convolution	128	5×5/2	80×60
	Dropout 2D P(x)=0.05	-		80×60
	Inception	(8, 4)	-	80×60
	Inception	(16, 8)	-	80×60
	Convolution	32	1×1	80×60
	Add	-	-	80×60
	Convolution	128	3×3/2	40×30
	Dropout 2D P(x)=0.025	-		40×30
	Inception	(8, 4)	-	40×30
	Inception	(16, 8)	-	40×30
	Inception	(32, 16)	-	40×30
	Convolution	64	1×1	40×30
	Add	-	-	40×30
	Convolution	256	3×3/2	20×20
VAE	Flatten	-	-	76 800
	Fully-Connected	-	-	512
	Mean	-	-	2
	Standard Deviation	-	-	2
	Sampling	-	-	2
Decoder	Fully-Connected	-	-	64
	Reshape	-	-	4×4×4
	Inception 3D	(32, 16)	-	4×4×4
	Inception 3D	(16, 8)	-	4×4×4
	Inception 3D	(8, 4)	-	4×4×4
	Convolution 3D	16	1×1×1	4×4×4
	Add	-	-	4×4×4
	Transposed Conv 3D	64	3×3×3×2	8×8×8
	Inception 3D	(16, 8)	-	8×8×8
	Inception 3D	(8, 4)	-	8×8×8
	Convolution 3D	16	1×1×1	8×8×8
	Add	-	-	8×8×8
	Transposed Conv 3D	32	3×3×3×2	16×16×16
	Inception 3D	(8, 4)	-	16×16×16
	Convolution 3D	16	1×1×1	16×16×16
	Add	-	-	16×16×16
	Transposed Conv 3D	4	5×5×5×2	32×32×32
	Convolution 3D (Softmax)	2	3×3×3	32×32×32

**Table 3:** Anchor scales in pixels calculated using the *K-Means* clustering method.

Anchor Type	Anchor 1	Anchor 2	Anchor 3
Small	18.83, 47.53	52.34, 37.53	34.13, 73.28
Medium	86.35, 46.02	62.74, 68.31	62.75, 102.19
Large	96.69, 84.20	103.66, 119.51	136.34, 146.64

**Table 4:** Mean precision values in respect to IoU>0.5 for each of our trained models.

Network Type	mAP (%)
Our RGB-D	60.20%
*YoloV3* RGB-D	55.75%
Our RGB	41.27%
*YoloV3* RGB	37.96%
Our Depth	26.46%
*YoloV3* Depth	20.87%

**Table 5:** Visual evaluation of object reconstruction. Table presents: RGB frame, original depth frame; reconstructed cloud of voxels; triangulated and smoothed surface created using predicted voxel cloud; and a corresponding similar object in the training set.

RGB	Depth	Voxel Cloud	Mesh	Training Data
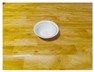	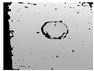	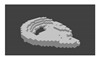	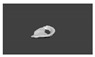	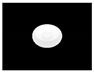
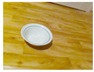	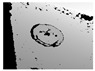	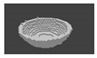	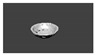	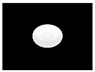
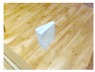	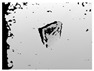	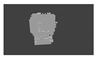	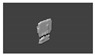	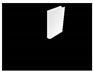
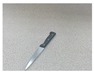	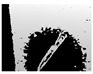	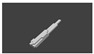	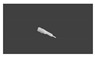	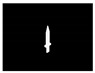
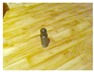	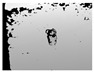	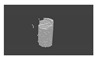	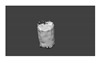	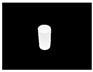
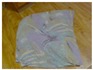	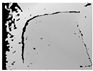	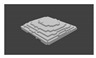	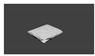	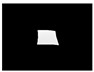
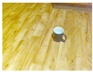	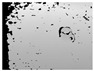	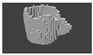	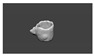	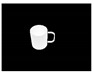
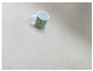	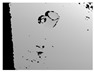	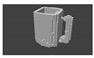	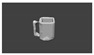	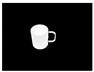
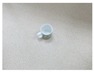	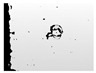	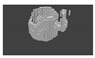	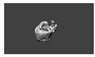	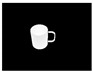
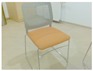	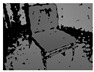	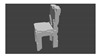	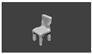	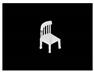
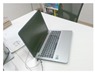	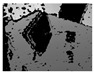	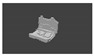	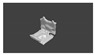	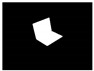
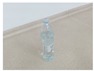	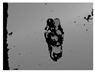	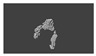	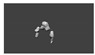	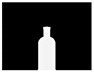

**Table 6:** Comparison of neural network complexity by the number of parameters, number of operations and model size.

Network Model	No. of Parameters	No. of Operations	Model Size (MB)
YOLOv3 [47]	61.81 M	294.86 M	946
Proposed (extended YOLOv3)	67.45 M	305.61 M	1010
AlexNet [79]	60 M	16.04 G	217
GoogleNet [80]	7 M	16.04 G	40
ResNet152 [81]	60 M	11.3 G	230
VGC16 [82]	138 M	154.7 G	512.24
NIN [83]	7.6 M	11.06 G	29
SimpleNet [84]	5.4 M	652 M	20
